# Functional Disruptions of the Brain in Low Back Pain: A Potential Imaging Biomarker of Functional Disability

**DOI:** 10.3389/fneur.2021.669076

**Published:** 2021-07-14

**Authors:** Bidhan Lamichhane, Dinal Jayasekera, Rachel Jakes, Wilson Z. Ray, Eric C. Leuthardt, Ammar H. Hawasli

**Affiliations:** ^1^Department of Neurosurgery, Washington University School of Medicine, St. Louis, MO, United States; ^2^Department of Biomedical Engineering, Washington University in St. Louis McKelvey School of Engineering, St. Louis, MO, United States; ^3^Meritas Health Neurosurgery, North Kansas City, MO, United States

**Keywords:** chronic low back pain, graph theory, support vector machine, feature selection, elastic net

## Abstract

Chronic low back pain (LBP) is one of the leading causes of disability worldwide. While LBP research has largely focused on the spine, many studies have demonstrated a restructuring of human brain architecture accompanying LBP and other chronic pain states. Brain imaging presents a promising source for discovering noninvasive biomarkers that can improve diagnostic and prognostication outcomes for chronic LBP. This study evaluated graph theory measures derived from brain resting-state functional connectivity (rsFC) as prospective noninvasive biomarkers of LBP. We also proposed and tested a hybrid feature selection method (Enet-subset) that combines Elastic Net and an optimal subset selection method. We collected resting-state functional MRI scans from 24 LBP patients and 27 age-matched healthy controls (HC). We then derived graph-theoretical features and trained a support vector machine (SVM) to classify patient group. The degree centrality (DC), clustering coefficient (CC), and betweenness centrality (BC) were found to be significant predictors of patient group. We achieved an average classification accuracy of 83.1% (*p* < 0.004) and AUC of 0.937 (*p* < 0.002), respectively. Similarly, we achieved a sensitivity and specificity of 87.0 and 79.7%. The classification results from this study suggest that graph matrices derived from rsFC can be used as biomarkers of LBP. In addition, our findings suggest that the proposed feature selection method, Enet-subset, might act as a better technique to remove redundant variables and improve the performance of the machine learning classifier.

## Introduction

Chronic low back pain (LBP) is a leading contributor to disability globally. In the United States, LBP is linked to higher healthcare and socioeconomic costs, including reduced employee productivity (1) and lost wages estimated at $100 billion in 2006 (2). Despite advancements in diagnostic and therapeutic technology, researchers and clinicians have found the clinical management of LBP challenging due to its complex pathophysiology (3). This could be attributed to the absence of significant abnormalities in modern spinal imaging of LBP patients (4). These findings have given impetus to the identification of noninvasive biomarkers that have the potential to facilitate early diagnoses, guide treatment plans, and improve our understanding of LBP progression and severity.

In the past, several putative non-imaging biomarkers have been investigated in LBP (5–7). However, these biomarkers are often invasive and do not assess the impact of LBP on the brain. Contrastingly, a neuroimaging biomarker for pain uses different imaging modalities to reveal underlying information about the anatomical circuity and functional pathways that form a signature for chronic pain (8). Functional magnetic resonance imaging (fMRI) is a popular imaging modality used to study functional interactions between brain regions based on the performance of a task (task-fMRI) (9, 10) or while at rest (11, 12). However, task-fMRI can present physical challenges for some LBP patients who are unable to perform the required tasks. Resting-state fMRI (rs-fMRI) is a suitable alternative modality in which spontaneous changes in the blood-oxygen level dependent (BOLD) signal are recorded to identify patterns of functional connectivity while the patient is at rest (13). In fact, rs-fMRI can be used to gain a better understanding of the organization of the brain's cognitive function (14) and overcome some of the limitations of task-fMRI (15).

Resting-state functional connectivity (rsFC) is commonly used as a noninvasive biomarker for various neurological conditions (16). Functional connectivity refers to the temporal dependence of patterns of neural activity in spatially distant regions of the brain (17–20). Past studies have shown that aberrant functional processing within certain brain regions can cause sustained, and sometimes amplified perception of pain (21). This is supported by a growing body of evidence across many chronic pain disorders (22–24) including LBP (25–28). Using novel methods when analyzing brain activity can reveal unique insights (for example, reorganization of hub activity) into chronic pain conditions (29–32). Graph theory measures can be used to model patterns of rsFC as nodes (cortical regions) and edges (functional connections between cortical regions), which can help outline the organization of brain networks. This approach enables us to analyze the topology of networks, revealing underlying information about the higher-order organization of brain networks (33). Many studies have investigated graph measures across chronic pain conditions such as knee pain (34), fibromyalgia (35), and neck pain (24). However, this approach has only rarely been used in practice for LBP (36).

It can be difficult to identify disruptions in functional connectivity, especially in chronic pain, as rsFC matrices tend to be multivariate in nature (37, 38). This problem can be addressed by using a machine learning classifier (39, 40). Classification learning algorithms can accurately predict an unseen test dataset by using a set of essential training features. However, redundant features need to be removed from the dataset by using an appropriate feature selection method to improve classification accuracy (41). Elastic Net (Enet) is a widely used feature selection method that eliminates redundant variables that affect prediction accuracy (42). Enet is especially favorable when the number of predictors is higher than the sample size or when there are many correlated predictor variables. However, certain feature sets selected by Enet may not always constitute a best performing subset of features, as removing additional redundant variables could increase the classifier's performance. Thus, there is an unmet need for an optimal feature selection method. To address this need, we proposed and tested a new hybrid feature selection approach which sorted features according to the magnitude of their Enet coefficients. The best subset of predictors to be retained in the final model was then determined by the maximum cross validated AUC of the feature set. This feature selection approach is the combination of Enet with an optimal subset selection extension which we refer to as Elastic Net-subset (or Enet-subset).

In summary, our group (36) and other researchers have shown that LBP patients present with disruptions in cortical functional connectivity. We have also shown that an SVM is capable of using variations in cortical thickness to classify LBP from HC (36). To further expand on our previous work, we (1) extracted local graph measures from functional connectomes and determined their ability to predict LBP by (2) testing a new hybrid feature selection technique (Enet-subset). We hypothesized that LBP patients would show differences in functional connectivity in previously implicated pain processing regions, which a machine learning classifier could use to predict patient group. We also examined if an Enet-subset feature selection approach could improve classifier performance by removing additional redundant variables. We collected high-resolution resting-state scans and parcellated the processed data using a multi-modal parcellation (MMP) developed by the Human Connectome Project (HCP) (43). We also collected self-reported clinical data for the Oswestry Disability Index (ODI) outcome measure.

## Methods and Materials

### Participants

The subjects who participated in this study included 27 healthy controls (HC) and 24 LBP subjects (age matched; *p* = 0.21). This study received approval from the Washington University in Saint Louis Institutional Review Board. All LBP subjects recruited for this study had been diagnosed with chronic LBP due to lumbar spondyloarthropathy with a history of 6 months without lower extremity symptoms. All LBP patients had not received lumbar spine surgery at the time of scanning. All HCs had no history of neurological injury or disease prior to their scan. Table 1 summarizes the participant information (refer Supplementary Material for inclusion and exclusion criteria).

**Table 1:** Participants' demographic information.

**Variable**	**Healthy controls**	**LBP**
Participants (*n*)	27	24
Sex (M/F)	15/12	9/15
Age (in years)	46.9 ± 17.3 (25–75)	53.5 ± 10.2 (29–67)

All participants were recruited through the Washington University School of Medicine Research Participant Registry (Volunteer for Health) and direct patient contact during clinical visits at the Barnes Jewish Hospital, Washington University School of Medicine, and Barnes Jewish West County Hospital. All participants were screened by a physician prior to enrollment in the study, and written informed consent was obtained prior to scanning.

### Clinical Data Acquisition

Data for the Oswestry Disability Index (ODI) questionnaire (44, 45) was collected from each participant. The ODI is considered the clinical “gold standard” for assessing functional disability in individuals with LBP (46). The ODI is a self-administered, 10-item questionnaire related to impairments like pain, and abilities such as standing, walking, traveling, lifting, socializing, sitting, personal care, sleeping, and sex life (45). Each item is scored from 0 to 5, and the total of the ten items is expressed as a percentage of the maximum score ranging from 0 (no disability) to 100 (maximum disability).

### fMRI Data Acquisition and Pre-processing

A 3T Siemens Prisma with a 32-channel head coil was used to collect 0.8 mm isotropic T1-weighted and T2-weighted scans from all participants. Resting state fMRI images were acquired on the same day using multi-band gradient echo EPI (multi-band accel. factor = 6). The scans had high spatial (2.4 mm × 2.4 mm × 2.4 mm) and temporal (TR = 800ms) resolution [repetition time (TR) = 800 ms, echo time (TE) = 33 ms and flip angle = 52°]. A 2.4 mm isotropic spin echo field map was also collected during fMRI acquisition to correct for any distortion in the fMRI data.

We collected six resting-state fMRI scans that were 5 min long, with AP/PA phase encoding directions (60 axial slices each). Volumetric navigator sequences were used to collect T1- and T2-weighted sequences that were corrected for motion by repeating scans (47). During the resting scans, subjects focused their attention on a visual crosshair.

The imaging data was preprocessed using the HCP's preprocessing pipelines (v4.0.0) (43, 48–51). The structural preprocessing pipelines were used to generate subcortical segmentations and cortical surfaces. Following structural pre-processing, the functional pre-processing pipelines corrected for EPI distortion, registered the fMRI data to structural MRI, and then brought the cortical time series from the volume dimension to the surface. The denoising pipelines then registered the fMRI data to the structural MRI data and corrected for motion and distortions within fMRI data by mapping it onto a CIFTI grayordinate space and removing spatially specific noise. The MSMAll areal-feature-based cross-subject surface registration pipeline was then applied to align the individual subject's cortical regions to the HCP's multi-modal parcellation. This process is more accurate than using cortical folding alone. Finally, temporal ICA (50, 52) was used to clean global noise from the MSMAll aligned rs-fMRI data. For this process, weighted regression (43) of group spatial ICA components from a much larger HCP-Young Adult 1,071-subject dataset with an existing temporal ICA decomposition was applied and the resulting concatenated individual subject time series were unmixed using the previously computed temporal ICA unmixing matrix. The noise temporal ICA individual subject component timeseries from this larger dataset were then non-aggressively regressed out from the subject's timeseries (see Supplementary Methods for more information on pre-processing methods). DVARS excursions were used to quantify patient movement (53) and revealed no statistically significant difference between the two patient groups.

### Graph Theory Analyses

**Figure 1:**
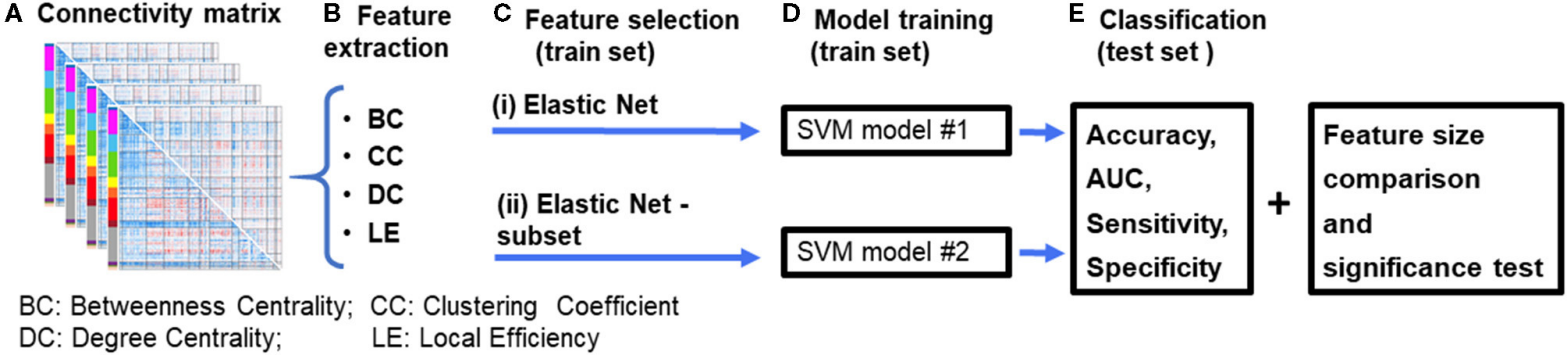
Diagrammatic representation of the data processing pipeline. **(A)** First, resting-state functional connectivity (rsFC) matrices are computed for each subject. **(B)** Graph theory features are then extracted from the connectivity matrices. **(C)** Features were selected using an (i) Elastic Net feature selection method, and (ii) proposed Elastic Net-subset (Elastic Net + optimal subset selection) approach to identify predictive features while reducing feature redundancy. **(D)** Two SVM models were constructed for each of the feature selection approaches. **(E)** Each model's performance (accuracy, AUC, sensitivity, specificity, and the total number of features used in the final model) were computed and then compared between both models. A significance test was performed using a permutation test approach. The whole process was repeated for each feature set and their combinations (for example, BC+CC+DC). BC, Betweenness Centrality; CC, Clustering Coefficient; DC, Degree Centrality; LE, Local Efficiency.

Nodes of the functional network were defined as one of 360 non-overlapping parcels from the HCP's MMP. We constructed functional connectivity matrices for each subject by taking the average timeseries of each of the 360 cortical regions from the pre-processed fMRI data. We then computed the Pearson's correlation coefficient for each pair of cortical regions before applying a Fisher-z transformation (Figure 1A). We then thresholded all graphs at the same network densities and binarized them to avoid biased graph metric comparisons between patient populations (54–56). Binarization is a very effective method of preserving the most probable functional connections (57, 58). Since there is no universally accepted threshold for functional connectivity strength, we decided to threshold connections within the top 15% by network density for each individual, in steps of 2.5% up to 30% density, to create binary undirected graphs for each density. The graph theory metrics were then averaged across these thresholds for each node (35, 36, 59).

We used the Brain Connectivity Toolbox (60) to calculate the following local graph measures for each patient: clustering coefficient, local efficiency, degree centrality, and betweenness centrality (Figure 1B). The *clustering coefficient* (the fraction of connected triangles in a network) measures the degree to which a node's neighbors are connected to each other (60). The *degree centrality* (the number of edges for a specific node) assumes that the importance of a node is related to the number of nodes that it is directly connected to Barabási and Albert (61). The *betweenness centrality* (a centrality measure based on shortest paths) is a measure of how influential a node is as information passes through it to other nodes (62). The *local efficiency* measures the efficiency of information transfer within the local neighborhood of a node (63). These metrics investigate network properties within the local neighborhood of a node and have been the subject of many studies across various chronic pain conditions (25, 34, 59).

### Machine Learning Classification

We used a support vector machine (SVM) with a linear kernel as a classifier in this study. The pool of subject data was randomly separated into training and testing sets in a 70/30 ratio, keeping the ratio of patients in each group constant (i.e., the ratio of HC to LBP). The training dataset was used for the feature selection (Figure 1C) and model training (Figure 1D) phases. The model's performance was tested using the testing dataset (Figure 1E). We used the caret and glmnet packages available in RStudio (64, 65) for our machine learning analysis.

#### Feature Selection

Each cortical parcel was modeled as a node such that 360 features were extracted for each graph theory measure. These features were then used in two different feature selection approaches that aimed to remove any redundant features to increase the classifier's performance and lead to better generalization of independent datasets. The first feature selection approach, Elastic Net (Enet), shrinks the coefficients of the input features to zero if they are not positively contributing. Parameter optimization was done by using a grid approach on the predefined penalty parameter λ = seq (0.1, 0.9, by =0.1] and α = seq ([0.0001, 0.005, by =0.001). We were constrained to a small alpha value due to the small number of features that survived (non-zero coefficients). In addition, we chose a small alpha value as increasing it would have led to underfitting the SVM classifier with this dataset. Following this, all the features with non-zero coefficients that form the Enet were used as the input to the SVM classifier (SVM model #1 in Figure 1D).

The second feature selection approach, Enet-subset, uses the coefficients estimated by Enet. The features were sorted in descending order based on the absolute values of the coefficients (Step #1). The sorted features were then used to build an SVM classifier (model #2, Figure 1D). We trained the classifier using a subset starting with the top 25 features, ranked by feature coefficient, with a step size of 25 (Step #2). The best subset of predictors retained in the final model was then determined by the maximum cross-validated AUC. The procedure for the Enet-subset method is summarized below:

***Step #1:*** Sort the absolute value of Enet coefficients in descending order.

***Step #2***: In a loop,

for, each subset = range [25: the total number of features, step size = 25].

AUC was computed for each subset using an SVM linear classifier and nested 4-fold cross-validation approach.

end

***Step #3*** The AUC was determined for all subsets, and the best performing subset (out of the subsets tested) was used in the final SVM (model #2, Figure 1D).

#### Model Training and Classification

In the model training phase, features selected using the Enet and Enet-subset methods were used to train two separate SVM models (SVM model #1 and SVM model #2, Figure 1D). As before, the features were normalized, and optimal model parameters were fed into each final SVM model. We used a grid-search algorithm to optimize the cost (C) of each SVM classifier. The search scale was set to C = 1:10, and the cost with the highest performance was used in each final model. To generalize the training process and obtain a more accurate model, we used a *K*-fold (*K* = 4) cross-validation, which was repeated five times. This technique divides data into equal disjointed subsets of size four. The model was then trained on all folds except one. The remaining subset was reserved for testing purposes. This process was then repeated three (*K*−1) times, selecting each fold to be used for testing once. We repeated this process five times to ensure that our trained model acquired most of the patterns from the training dataset.

We evaluated the performance of each SVM model using the test dataset where HCs were classified as positive and LBP as negative for the true positive (TP), false positive (FP), true negative (TN), and false negative (FN) calculations. We determined the corresponding accuracy, sensitivity, and specificity of each model. The accuracy (%) is defined as the fraction of correctly classified subjects {(TP+TN)/(TP+TN+FP+FN)}. Sensitivity is defined as the fraction of correctly classified positive samples from all positive samples {TP/(TP+FN)}. Specificity is defined as the fraction of correctly classified negative samples from all negative samples {TN/(TN+FP)}. We then determined the area under the receiver operating characteristics curve (AUC) to evaluate each model's overall performance.

### Statistical Tests

We used an unpaired two-sample Wilcoxon rank-sum test to determine any statistically significant (*p* < 0.05) relationships in graph measures between both patient groups. We corrected for multiple comparisons by using False Discovery Rate Correction with *q* < 0.05.

During the model training phase, the data was randomly divided into testing and training datasets which may produce slightly different models depending on the division. To address this, the SVM was run 100 times (Figures 1C–E) and the results were averaged to calculate final performance measures. The arithmetic mean of the accuracy, sensitivity, specificity, and AUC of the 100 repetitions was computed for the final analysis.

Statistical significance of the classification accuracy and AUC were tested using permutation testing with 1,000 permutations. For this step, the subject's class (group) was randomly assigned. The resulting accuracy produced a null-hypothesis distribution that was then used to calculate the *p*-value of the corresponding accuracy (i.e., the fraction of permutations that produced a greater accuracy than the accuracy found for the classification models) (66).

## Results

### Clinical Survey Data

We used a Wilcoxon rank-sum test to compare the total ODI outcome scores from LBP patients to those from HC (Table 2). There was a significant difference (*p* = 7.21e-9; *z* = 5.79) in the total ODI scores of LBP and HCs. Patients with chronic LBP had a higher total ODI score which was indicative of higher functional disability.

**Table 2:** ODI scores for each patient group.

**Patient group**	**Mean**	**Median**	**Standard deviation**
Low back pain	33.3	34.0	15.3
Healthy controls	5.63	6.00	5.60

### Differences in Graph Metrics Between LBP and HC

Our analysis showed no significant differences in the local efficiency (LE), clustering coefficient (CC), degree centrality (DC), and betweenness centrality (BC) of individual nodes from the reconstructed brain networks between LBP patients and HCs after FDR correction (all *p* > 0.05, see Supplementary Table 1). While this may be unexpected, some studies have shown that chronic pain states show no univariate associations with local graph measures (67). A non-significant group difference in a univariate analysis does not necessarily imply a weak feature in a multivariate machine learning analysis approach (68). In fact, a univariate analysis is often less comprehensive than a multivariate model and is unable to show relationships between multiple variables (or parcels) (69, 70). We demonstrate (Supplementary Figures 1, 2) that, while not significant, multiple parcels show differences in graph metrics between LBP and HCs. A multivariate approach can use these differences to chart meaningful relationships. In addition, we use feature selection to discard noisy features and reduce the number of features. For these reasons, we used a multivariate approach with an SVM to overcome the shortcomings of univariate analyses.

### Machine Learning to Predict LBP

We used the BC, CC, DC, and LE of all 360 parcels to train an SVM to correctly predict each subject's patient group (see section Graph Theory Analyses) and determine the matrix of best performing features for each graph measure. Of the four graph theory matrices used, BC, CC, and DC had very high classification accuracies when used on their own with both feature selection approaches. However, LE proved to have a low classification accuracy with both feature selection approaches. We repeated our analyses to determine if a combination of graph measures led to a higher classification accuracy than a single measure. We then combined the BC, CC, and DC datasets, and compared their predictive power between the two feature selection methods. In all iterations, the performance of the classifier increased when using Enet-subset features (except for LE). We achieved a maximum (mean of 100 iterations) classification accuracy of 83.1% (*p* < 0.004), AUC of 0.94 (*p* < 0.002), sensitivity of 87 % (*p* < 0.076), and a specificity of 79.7% (*p* < 0.054) when using BC, CC, and DC with an Enet-subset feature selection approach. Table 3 summarizes the overall classification results (see Supplementary Table 2 for the sensitivity and specificity of each model using each feature selection method).

**Table 3:** A summary (mean of 100 iterations) of the classification accuracy and AUC using the Enet and proposed Enet-subset feature selection methods.

**Biomarker(s)**	**Using all Enet selected features**		**Using Enet-subset selected features**	
	**ACC (%), AUC (mean)**	**Features (mean/total #)**	**ACC (%), AUC (mean)**	**Features (mean/total #)**
BC	81.7, 0.919	349/360	82.6, 0.920	326/360
CC	81.0, 0.92	349/360	82.3, 0.925	328/360
DC	80.9, 0.898	348/360	81.2, 0.895	324/360
LE	50.8, 0.598	348/360	50.4, 0.590	155/360
BC+CC	81.0, 0.923	679/720	82.5, 0.92	634/720
BC+DC	81.2, 0.907	680/720	83.2, 0.924	636/720
CC+DC	80.8, 0.913	680/720	81.8, 0.921	640/720
BC+CC+DC	80.9, 0.916	1,006/1,080	83.1, 0.937	945/1,080

*ACC, Accuracy; AUC, Area under curve; BC, Betweenness centrality; CC, Clustering coefficient; DC, Degree centrality; LE, Local efficiency*.

We saw that the Enet-subset feature selection method was successful in reducing the total number of selected features used in the final models. As a result, the prediction accuracy of the proposed Enet-subset feature selection approach is higher in all instances when compared to using Enet as a baseline (except for LE). This supports our hypothesis that the Enet-subset method performs better at removing redundant features (i.e., fewer noisy features results in a higher model accuracy). This effect is most noticeable when the total number of features used is relatively large (for example, using 360 features from BC vs. using 1,080 features by combining features from BC+CC+DC) while also having the best classifier performance of all the models tested.

### Frequently Selected Features

**Figure 2:**
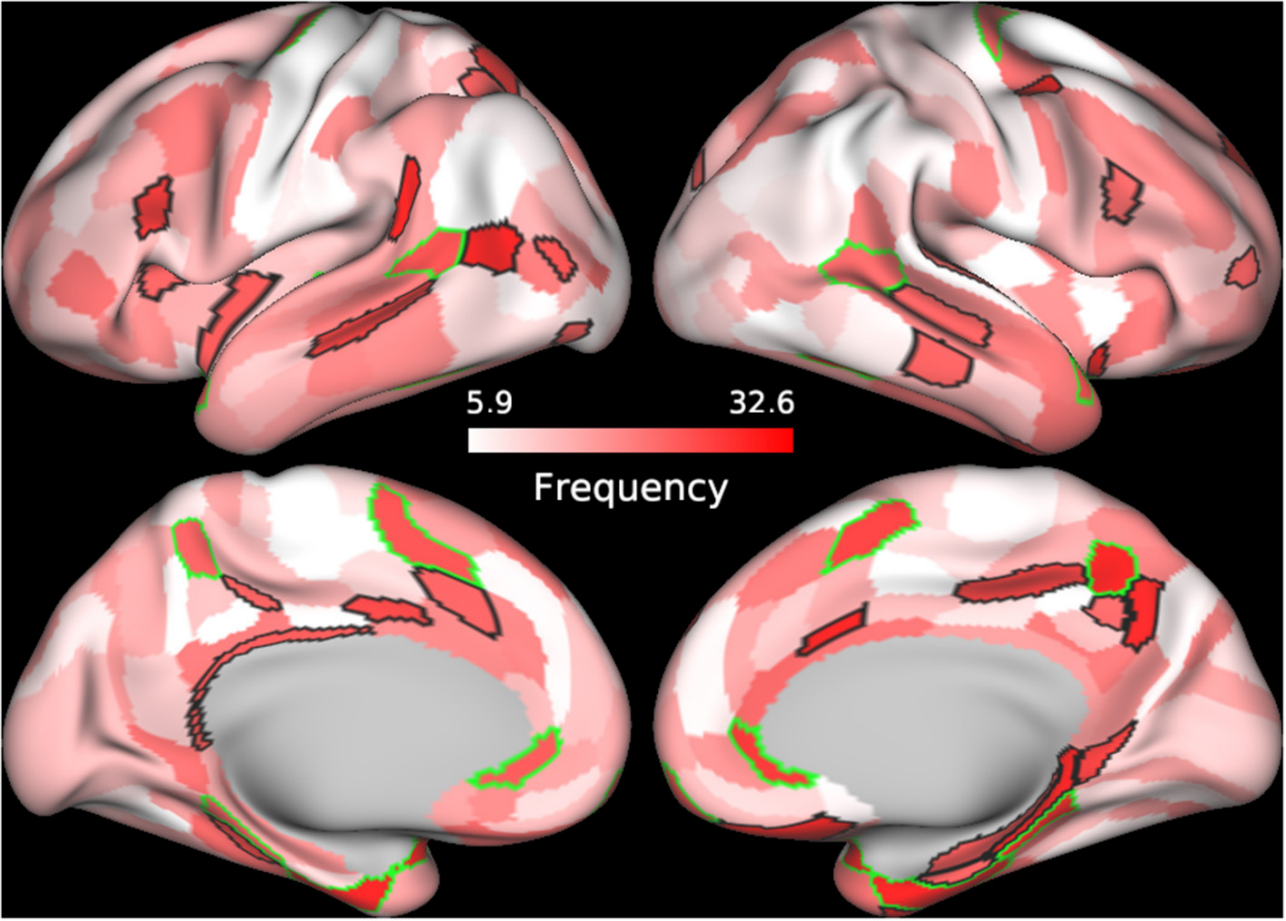
Frequently selected features. The frequency of selection for each cortical feature used to train the SVM model using BC+CC+DC and proposed Enet-subset feature selection method was plotted onto a cortical mesh surface. The top 60 features were selected in all 100 iterations and sorted according to the frequency of its selection during the 100 iterations. Cortical regions outlined in green are bilateral while those outlined in black are unilateral.

In order to further understand the role of individual parcels in the classification, we identified the top 60 cortical regions (ranked by frequency) of the best performing SVM classifier in which BC, CC, and DC were used as features and Enet-subset was used for feature selection during each iteration. We then sorted the cortical regions according to their frequency of repetition. The top 60 frequently selected cortical regions that contributed to the classification were plotted on a brain mesh surface based on a scale corresponding to their frequency values (Figure 2, see Supplementary Table 3 details on individual areas). In addition, we plotted the top 60 frequently selected cortical regions that contributed to the classification of each individual graph measure (see Supplementary Figures 3–5 and Supplementary Tables 4–6 for more details on individual parcels).

We also conducted a Pearson's correlation to determine any correlations between the graph measures of the top 60 frequently selected cortical parcels (Figure 2 and Supplementary Table 3) and the patient's corresponding total ODI scores. However, we did not find any significant correlations between these graph measures and the calculated total ODI scores.

## Discussion

The literature has shown that a high level of functional interaction between cortical regions is necessary to cope with the demand of cognitive activities (71–73). We used noninvasive imaging in this study to model these functional interactions and measure network properties. This study builds on our previous work (36) by using graph theory metrics in the classification process to understand a complementary component of cortical changes in the LBP syndrome. The results from this study (1) validate our hypothesis that the use of certain graph measures as a biomarker may lead to the integration of more effective information on pain states like LBP and (2) support the Enet-subset method as a more effective feature selection algorithm for removing redundant variables and improving the classifier's performance. In addition, we found graph measures to be very accurate predictors of patient group irrespective of the feature selection technique used. The success we have seen with the machine learning models supports the notion that groups of cortical regions are more predictive of the patient group than individual cortical regions.

### Predictive Cortical Regions Are Involved in Spatio-Temporal Processing and Its Associated Visual and Motor Coordination

**Figure 3:**
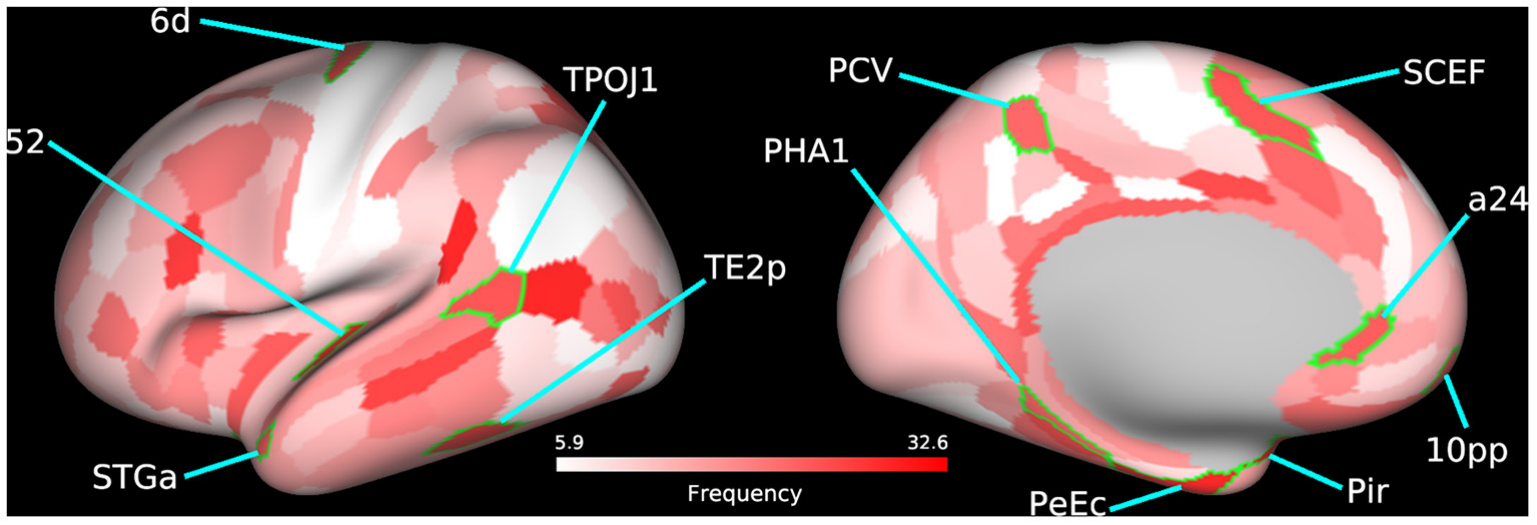
Bilateral frequently selected features. Bilateral cortical regions from the 60 most frequently selected parcels used to train the SVM model using BC+CC+DC and an Enet-subset feature selection method are highlighted on a cortical mesh surface of the left hemisphere. Right hemisphere is not shown. Cortical regions are outlined in green and labeled according to the abbreviations in Table 4. Frequency of selection is indicated in red.

The temporal-parietal-occipital junction (TPOJ), precuneus visual area, supplementary and cingulate eye field (SCEF), parahippocampal area (PHA), and perirhinal cortex are some key bilateral cortical regions (Figure 3; Table 4; Supplementary Figure 6) that were frequently selected as predictive features and are involved in spatial navigation. Spatial navigation is a resource-demanding process that involves determining and maintaining an optimal trajectory to a target based on incoming sensory stimuli from surrounding spatial references (74).

**Table 4:** A summary of the bilateral regions from the top 60 cortical regions, selected for by frequency, that contributed to the classification accuracy of the Enet-subset model when trained using the betweenness centrality, degree centrality, and clustering coefficient graph measures.

**Area name**	**Area description**
PCV	Precuneus visual area
SCEF	Supplementary and cingulate eye field
6d	Dorsal area 6
a24	Area a24
10pp	Polar 10p (Orbitofrontal cortex)
52	Area 52 (Parainsular area)
Pir	Pirform cortex (Olfactory)
PeEc	Perirhinal ectorhinal cortex
STGa	Area STGa (auditory)
PHA1	Parahippocampal area 1
TE2p	Area TE2 posterior
TPOJ1	Area TemporoParietoOccipital Junction 1

The TPOJ has been implicated in numerous functions (75, 76) such as attentional reorienting between spatial locations (77), timing of visual events (78), visual awareness (79), and the integration of these different sensory inputs (80, 81). The precuneus visual area plays an important role in spatial navigation (82) and spatial processing (83). Previous studies have shown that damage to this part of the parietal cortex leads to deficits in spatial representational (84), simultagnosia (85), and oculomotor apraxia (86), all of which are related to visuospatial processing. These findings suggest that the precuneus may likely be involved with how we interpret external events as painful but not directly involved in the cortical representation of pain (87).

The SCEF is a part of the supplementary motor complex that is associated with the regulation of eye movement (88). The SCEF has anatomical connections to the frontal eye field, superior colliculus, and lateral intraparietal cortex, which puts it in a unique position to regulate goal-directed behavior (89, 90). Dorsal area 6 is a part of the dorsal premotor cortex (DPC), which is also implicated in goal-directed actions involving target object, hand, and eye positioning (91). Inhibiting activity of the DPC using transcranial magnetic stimulation in human patients increases reaction times which supports its role in motor planning (92). These findings are bolstered by the significant increase in functional disability in LBP patients as shown by the differences in ODI scores between both patient groups.

The PHA is a subregion of the ParaHippocampal cortex (PHC) reported to be involved in visuospatial processing (93), including place perception (94), and spatial representation (95, 96). Individuals with lesions to the PHC show impaired visuospatial processing and difficulties with spatial orientation, navigation, and landmark identification (97, 98). Area a24, a part of the anterior cingulate cortex (ACC), has been reported to show vestibular activations (99, 100). In addition, there is growing evidence that spatial memories may become supported by certain extrahippocampal structures over time. The ACC is believed to be one of these structures that stores past spatial memories (101).

The perirhinal cortex adds semantic knowledge to aid in item identification (102). In addition, the perirhinal cortex integrates item information with spatio-temporal information and transmits this data to the hippocampus *via* the entorhinal cortex (103). The temporal area 2 posterior (TE2p) is a newly identified cortical area that lies on the inferior temporal gyrus (43) and may play a role in visual pathways, specifically object recognition.

These bilaterally affected regions are engaged in the coordination of motor control and other sensory processes that facilitate spatial navigation. Studies have shown that physical self-awareness and perception of one's relative position is impaired in patients with severe chronic LBP (104, 105). Our previous work on this LBP population also found several cortical regions involved in spatial navigation to be predictive of patient group when trained using variations in cortical thickness (36). This evidence compounded by the downstream hand and shoulder motor deficits, as shown by differences in patient ODI scores, further supports the predictive features selected by our model. The identified regions could therefore serve as putative therapeutic biomarkers of functional motor disability.

### Feature Selection Using Enet-Subset

Embedded feature selection is a popular feature selection technique, as it incorporates feature selection into the machine learning algorithm (106). The Least Absolute Shrinkage and Selection Operator (LASSO) (107) is a common embedded method used to identify a small number of informative features (108). This is because of its ability to zero the coefficients of non-informative features and assign positive or negative coefficients to more informative features. However, the maximum number of features that LASSO can select is less than the total sample size. As a result, LASSO is an ineffective option when many features are required to train the classifier. We encountered this problem with our dataset when applying LASSO. In many of its iterations (out of 100), LASSO selected very few features even after optimizing the penalty parameter (λ). This led to the underfitting of our models resulting in a poor model performance. For this reason, we did not use LASSO in our final analysis.

We then applied Enet (42), an embedded feature selection method based on a relatively sparse model, to select for significant variables within each graph measure. However, it was apparent that Enet still selected redundant variables. Therefore, the performance of the model could be further improved by removing such variables. This was clearly seen when models trained using features selected by the Enet-subset feature selection method performed better with fewer features than when using Enet. These redundant variables need to be removed to increase the accuracy of the classifier. Redundant variables also lead to overfitting and an increase in calculation load which is computationally expensive. The proposed Enet-subset method further selects for significant variables using the optimal subset selection extension based on the feature's coefficient following Enet. As a result, the Enet-subset method is capable of reducing additional non-informative features. Therefore, the Enet-subset method is effective in reducing model complexity and calculation load with complex neuroimaging data. By using fewer features with the Enet-subset method, we improved the accuracy, AUC, sensitivity, and specificity of all models (see Table 3 and Supplementary Table 2). This would be another useful feature for large neuroimaging datasets.

## Limitations

There are several limitations in this study. We did not explore the cerebellum or subcortical regions, as the HCP's MMP does not parcellate these regions. The subcortical regions of the brain and cerebellum have been shown to play an important role in the coordination and control of movement and balance. Future studies should include these regions of the brain in their analysis for a more comprehensive outlook. In addition, these studies should also investigate the classification accuracy of lesser researched graph metrics such as *K*-coreness, flow coefficient, and participation coefficient.

Although our study shows that graph measures are of promising clinical value in predicting pain, there are some limitations mainly due to sample size. Therefore, our results should be considered with due caution. A suitable next step would include testing these models with a large sample and using regression models instead of classification models (for example—prediction of clinical pain and emotional measures which would help understand the progression and severity of the pain). It is also important to note that the validation of a biomarker would require testing its efficacy in identifying a disease state in the presence of other disease states. Therefore, future studies should replicate this approach with a sample population that includes other chronic pain states in addition to LBP. It is also important to note that pain is a multidimensional process which involves multiple brain networks interacting with each other. This can present challenges when interpreting the functional role of cortical regions and should be considered with care.

Chronic LBP is a syndrome that presents with numerous etiologies and varying symptomatology. Therefore, our attempts to recruit a homogenous population of subjects without a history of spine surgery were met with difficulty. Finally, some potential pitfalls that could arise from the machine learning methods include incomplete, biased data or noisy datasets and overfitting. These drawbacks could be addressed by recruiting larger matched samples and testing the models on more unseen data.

## Conclusion

In conclusion, the highly predictive graph theory network approach used to train the classifiers support the notion of brain function alteration in LBP. Our results demonstrate that machine-assisted classification algorithms can accurately classify patients into their respective cohort using graph theory metrics. This supports our hypothesis that these graph measures can be used as a biomarker of LBP. Our results also show that an Enet-subset feature selection method is more effective when improving a model's performance.

## Data Availability Statement

The datasets presented in this article are not readily available because, per the guidelines of the Institutional Review Board, they cannot be shared with individuals not included as research members on the project. Requests to access the datasets should be directed to Dinal Jayasekera, dinal.jayasekera@wustl.edu.

## Ethics Statement

The studies involving human participants were reviewed and approved by Washington University in St. Louis Institutional Review Board. The patients/participants provided their written informed consent to participate in this study.

## Author Contributions

BL and DJ: conceptualization, data collection, methodology, investigation, data analysis, visualization, and writing original draft. AH: conceptualization, data curation, funding acquisition, supervision, resources, and software. RJ: data analysis and writing original draft. WR and EL: supervision. All authors provided validation and helped with reviewing and editing the manuscript.

## Conflict of Interest

The authors declare that the research was conducted in the absence of any commercial or financial relationships that could be construed as a potential conflict of interest.
