# Supplementary material for: Functional Disruptions of the Brain in Low Back Pain: A Potential Imaging Biomarker of Functional Disability
Source: Front Neurol. 2021 Jul 14;12:669076. doi: 10.3389/fneur.2021.669076 (PMC8317987; doi:10.3389/fneur.2021.669076)
Supplement: Supplementary file 1 [file Data_Sheet_1.PDF]

Supplementary Methods, Tables and Figures For

Functional disruptions of the brain in low back pain: a potential imaging biomarker of functional disability

Bidhan Lamichhane[1]*, Dinal Jayasekera[2]*, Rachel Jakes[2], Wilson Z. Ray[1,2], Eric C. Leuthardt[1,2], Ammar H. Hawasli[1,3]

Affiliations:

[1]Department of Neurosurgery, Washington University School of Medicine, St. Louis, MO, 63110, USA.

[2]Department of Biomedical Engineering, Washington University in St. Louis McKelvey School of Engineering, St. Louis, MO, 63130, USA.

[3]Meritas Health Neurosurgery, North Kansas City, 64116, USA.

*These authors contributed equally to this work.

Supplementary Methods

Human Connectome Minimal Preprocessing Pipelines

The HCP *PreFreeSurfer* structural pipeline creates an undistorted structural volume space for each subject in which the T1- and T2-weighted images are aligned. A modified *FreeSurfer* pipeline segments MRI volumes into predefined structures and reconstructs cortical surfaces. The *PostFreeSurfer* pipeline then performs initial folding-based surface registration to an atlas using MSMSulc, computes T1w/T2w myelin maps and curvature-corrected cortical thickness maps and produces MRI volume and surface files that can be viewed on Connectome Workbench software and prepared for further analysis.

After the structural HCP pipeline is completed, functional pre-processing pipelines begin working on the individual time series files. The *fMRIVolume* pipeline removes EPI distortion, spatially realigns data for motion, registers fMRI data to structural MRI, and corrects the intensity bias field. The fMRISurface pipeline brings the cortical time series from the volume onto the surface and subcortical areas into alignment with MNI space based on nonlinear volume registration to form the grayordinate space. The multi-run spatial ICA+FIX pipeline demeans, detrends, and concatenates the subject's six fMRI runs before proceeding to remove spatially specific structured noise (from subject motion physiology and the scanner) from the fMRI data. MSMSulc is used to project the fMRI data onto the 32k

mesh before running MSMAll. The MSMAll surface-registration pipeline aligns cortical areas across subjects more precisely than is possible with cortical folding alone.

Temporal ICA (Glasser et al., 2019, 2018) was used to clean the MSMAll aligned resting state fMRI data of global noise after spatial ICA had been used to clean the data of spatially specific noise (using hand classification of spatial ICA components given that FIX performance on this 2.4mm dataset was 97%, indicating that FIX retraining was needed). Because of the relatively small size of the dataset, temporal ICA was unable to isolate a single or few global group noise components and instead found many single/few subject global components with imperfect separation of global signal and noise. Thus, instead of estimating the temporal ICA decomposition on this dataset, weighted regression (Glasser et al., 2016) of group spatial ICA components from a much larger HCP-Young Adult 1071-subject dataset with an existing temporal ICA decomposition was applied and the resulting concatenated individual subject component time courses were unmixed using the previously computed temporal ICA unmixing matrix. The noise temporal ICA individual subject component timeseries from this larger dataset were then non-aggressively regressed out from the subject timeseries producing similar resting state cleanup results to that which were previously published (Glasser et al., 2019, 2018).

Patient Inclusion and Exclusion Criteria

Lower back pain (LBP) patients in the study were recruited by running reports through Epic looking for a history of LBP over 6 months without lower extremity symptoms. Exclusion criteria include the following: ≤17 or >80 years old; pregnant; having an MRI-incompatible device; dental implants; amyotrophic lateral sclerosis, multiple sclerosis, rheumatoid arthritis, spine tumor, brain tumor, encephalopathy, traumatic brain injury, psychiatric disease, dementia, meningitis, previous incident of SCI, or HIV-related myelopathy; having systemic instability or being deemed unable to tolerate standard MRI scanning; abnormal orientation and cranial nerve physical examination. Patients with documented learning disabilities or patients who did not undergo standard of care post-injury physical therapy were excluded.

Supplementary Tables

Supplementary Table 1 – Statistical Significance of global graph theory measures

**Metric**	**LBP (mean ± SD)**	**HC (mean ± SD)**	**Statistic (mean ± standard deviation)**
**Betweenness Centrality**	456.97 ± 568.86	453.94 ± 576.74	z = 0.012 ± 0.243; p = 1
**Clustering Coefficient**	0.586 ± 0.194	0.585 ± 0.194	z = 0.0135 ± 0.28; p = 1
**Degree Centrality**	46.238 ± 30.423	46.798 ± 31.589	z = -0.0134 ± 0.378; p = 1
**Local Efficiency**	0.742 ± 0.222	0.745 ± 0.209	z = 0.167 ± 1.01; p = 0.485 ± 0.295

The p values shown have been corrected for multiple comparisons.

Supplementary Table 2 – Sensitivity and Specificity of Enet and Enet-subset feature selection approaches

Biomarker(s)	Using all Enet selected features		Using Enet-subset selected features	
	**SEN (%), SPE (%) (mean)**	**Features (mean / total #)**	**SEN (%), SPE (%) (mean)**	**Features (mean / total #)**
BC	85.4, 78.5	349/360	85.0, 80.6	326/360
CC	84.0, 78.4	349/360	84.2, 80.5	328/360
DC	82.5, 79.5	348/360	83.0, 79.6	324/360
LE	28.5, 70.5	348/360	42.0, 57.8	155/360
BC+CC	85.6, 77.0	679/720	85.2, 80.1	634/720
BC+DC	84.8, 78.0	680/720	85.8, 81.0	636/720
CC+DC	84.1, 78.0	680/720	82.7, 81.1	640/720
BC+CC+DC	85.2, 77.1	1006/1080	87.0, 79.7	945/1080

A summary (mean of 100 iterations) of sensitivity and specificity using the Enet and Enet-subset feature selection methods. SPE: Specificity; SEN=Sensitivity; BC: Between centrality; CC: Clustering coefficient; DC: Degree centrality; LE: Local efficiency.

Supplementary Table 3 – Top 60 cortical areas contributing to classification accuracy of BC, CC and DC combined

Parcel Number	Area Name	Area Description	Resting State Network	Hemisphere
12	55b	Area 55b	Language	R
14	RSC	RetroSplenial Complex	Frontoparietal	L
16	V7	Seventh Visual Area	Secondary Visual	R
22	PIT	Posterior InferoTemporal Complex	Secondary Visual	L
23	MT	Middle Temporal Area	Secondary Visual	L
25	PSL	PeriSylvian Language Area	Cingulo-Opercular	L
27	PCV	PreCuneus Visual Area	Posterior-Multimodal	R
-	-	-	Posterior-Multimodal	L
30	7m	Area 7m	Default Mode	R
38	23c	Area 23c	Cingulo-Opercular	R
43	SCEF	Supplementary and Cingulate Eye Field	Cingulo-Opercular	R
-	-	-	Cingulo-Opercular	L
50	MIP	Medial IntraParietal Area	Dorsal Attention	L
54	6d	Dorsal area 6	Somatomotor	R
-	-	-	Somatomotor	L
57	p24pr	Area Posterior 24 prime	Cingulo-Opercular	L
59	a24pr	Anterior 24 prime	Cingulo-Opercular	R
60	p32pr	Area p32 prime	Cingulo-Opercular	L
61	a24	Area a24	Default Mode	R
-	-	-	Default Mode	L
71	9p	Area 9 Posterior	Default Mode	R
79	IFJa	Area IFJa	Language	R
81	IFSp	Area IFSp	Frontoparietal	L
85	a9-46v	Area anterior 9-46v	Frontoparietal	R
90	10pp	Polar 10p	Default Mode	R
-	-	-	Default Mode	L
93	OFC	Orbital Frontal Complex	Default Mode	R
103	52	Area 52	Auditory	R
-	-	-	Auditory	L
106	PoI2	Posterior Insular Area 2	Cingulo-Opercular	L
107	TA2	Area TA2	Auditory	R
108	FOP4	Frontal OPercular Area 4	Cingulo-Opercular	L
110	Pir	Pirform Cortex	Orbito-Affective	R
-	-	-	Orbito-Affective	L
112	AAIC	Anterior Agranular Insula Complex	Orbito-Affective	R
118	EC	Entorhinal Cortex	Default Mode	R
119	PreS	PreSubiculum	Default Mode	R
120	H	Hippocampus	Default Mode	R
121	ProS	ProStriate Area	Primary Visual	R

122	PeEc	Perirhinal Ectorhinal Cortex	Ventral-Multimodal	R
-	-	-	Ventral-Multimodal	L
123	STGa	Area STGa	Language	R
-	-	-	Language	L
124	PBelt	ParaBelt Complex	Auditory	R
126	PHA1	ParaHippocampal Area 1	Default Mode	R
-	-	-	Default Mode	L
129	STSdp	Area STSd posterior	Language	R
130	STSvp	Area STSv posterior	Default Mode	L
136	TE2p	Area TE2 posterior	Dorsal Attention	R
-	-	-	Dorsal Attention	L
139	TPOJ1	Area TemporoParietoOccipital Junction 1	Language	R
-	-	-	Language	L
140	TPOJ2	Area TemporoParietoOccipital Junction 2	Posterior-Multimodal	L
145	IP1	Area IntraParietal 1	Frontoparietal	L
155	PHA2	ParaHippocampal Area 2	Default Mode	L
161	31pd	Area 31pd	Default Mode	R
162	31a	Area 31a	Frontoparietal	L
172	TGv	Area TG Ventral	Language	R
174	LBelt	Lateral Belt Complex	Auditory	R
177	TE1m	Area TE1 Middle	Default Mode	R

Supplementary Table 4 – Top 60 cortical areas contributing to classification accuracy of BC

Parcel Number	Area Name	Area Description	Resting State Network	Hemisphere
2	MST	Medial Superior Temporal Area	Secondary Visual	L
3	V6	Sixth Visual Area	Secondary Visual	R
4	V2	Second Visual Area	Secondary Visual	R
14	RSC	RetroSplenial Complex	Frontoparietal	L
16	V7	Seventh Visual Area	Secondary Visual	R
20	LO1	Area Lateral Occipital 1	Secondary Visual	L
23	MT	Middle Temporal Area	Secondary Visual	L
24	A1	Primary Auditory Cortex	Auditory	L
25	PSL	PeriSylvian Language Area	Cingulo-Opercular	L
27	PCV	PreCuneus Visual Area	Posterior-Multimodal	R
30	7m	Area 7m	Default Mode	R
33	v23ab	Area ventral 23 a+b	Default Mode	R
38	23c	Area 23c	Cingulo-Opercular	R
47	7PC	Area 7PC	Somatomotor	R
50	MIP	Medial IntraParietal Area	Dorsal Attention	L
54	6d	Dorsal area 6	Somatomotor	R
-	-	-	Somatomotor	L
59	a24pr	Anterior 24 prime	Cingulo-Opercular	R
60	p32pr	Area p32 prime	Cingulo-Opercular	L
61	a24	Area a24	Default Mode	R
63	8BM	Area 8BM	Frontoparietal	R
70	8BL	Area 8B Lateral	Default Mode	L
71	9p	Area 9 Posterior	Default Mode	R
76	47l	Area 47l (47 lateral)	Default Mode	L
81	IFSp	Area IFSp	Frontoparietal	L
85	a9-46v	Area anterior 9-46v	Frontoparietal	R
101	OP1	Area OP1/SII	Somatomotor	R
103	52	Area 52	Auditory	L
107	TA2	Area TA2	Auditory	R
-	-	-	Auditory	L
108	FOP4	Frontal OPercular Area 4	Cingulo-Opercular	L
110	Pir	Pirform Cortex	Orbito-Affective	R
112	AAIC	Anterior Agranular Insula Complex	Orbito-Affective	R
121	ProS	ProStriate Area	Primary Visual	R
-	-	-	Primary Visual	L
122	PeEc	Perirhinal Ectorhinal Cortex	Ventral-Multimodal	R
-	-	-	Ventral-Multimodal	L
123	STGa	Area STGa	Language	R

-	-	-	Language	L
125	A5	Auditory 5 Complex	Language	R
126	PHA1	ParaHippocampal Area 1	Default Mode	R
130	STSvp	Area STSv posterior	Default Mode	L
131	TGd	Area TG dorsal	Default Mode	R
132	TE1a	Area TE1 anterior	Default Mode	L
135	TF	Area TF	Ventral-Multimodal	R
136	TE2p	Area TE2 posterior	Dorsal Attention	R
-	-	-	Dorsal Attention	L
140	TPOJ2	Area TemporoParietoOccipital Junction 2	Posterior-Multimodal	L
142	DVT	Dorsal Transitional Visual Area	Primary Visual	L
145	IP1	Area IntraParietal 1	Frontoparietal	L
149	PFm	Area PFm Complex	Frontoparietal	L
156	V4t	Area V4t	Secondary Visual	R
161	31pd	Area 31pd	Default Mode	L
164	25	Area 25	Default Mode	L
165	s32	Area s32	Default Mode	L
172	TGv	Area TG Ventral	Language	R
176	STSva	Area STSv anterior	Default Mode	R
177	TE1m	Area TE1 Middle	Default Mode	R
-	-	-	Frontoparietal	L
180	p24	Area posterior 24	Cingulo-Opercular	R

Supplementary Table 5 – Top 60 cortical areas contributing to classification accuracy of CC

Parcel Number	Area Name	Area Description	Resting State Network	Hemisphere
10	FEF	Frontal Eye Fields	Cingulo-Opercular	R
12	55b	Area 55b	Language	R
13	V3A	Area V3A	Secondary Visual	L
16	V7	Seventh Visual Area	Secondary Visual	L
23	MT	Middle Temporal Area	Secondary Visual	L
25	PSL	PeriSylvian Language Area	Cingulo-Opercular	L
27	PCV	PreCuneus Visual Area	Posterior-Multimodal	R
29	7Pm	Medial Area 7P	Frontoparietal	R
33	v23ab	Area ventral 23 a+b	Default Mode	L
34	d23ab	Area dorsal 23 a+b	Default Mode	L
39	5L	Area 5L	Somatomotor	L
43	SCEF	Supplementary and Cingulate Eye Field	Cingulo-Opercular	L
45	7Am	Medial Area 7A	Cingulo-Opercular	R
49	VIP	Ventral IntraParietal Complex	Secondary Visual	L
54	6d	Dorsal area 6	Somatomotor	L
57	p24pr	Area Posterior 24 prime	Cingulo-Opercular	L
59	a24pr	Anterior 24 prime	Cingulo-Opercular	R
61	a24	Area a24	Default Mode	L
64	p32	Area p32	Default Mode	R
73	8C	Area 8C	Frontoparietal	L
74	44	Area 44	Language	R
75	45	Area 45	Language	R
79	IFJa	Area IFJa	Language	R
81	IFSp	Area IFSp	Frontoparietal	L
83	p9-46v	Area posterior 9-46v	Frontoparietal	R
85	a9-46v	Area anterior 9-46v	Frontoparietal	R
89	a10p	Area anterior 10p	Frontoparietal	R
-	-	-	Frontoparietal	L
90	10pp	Polar 10p	Default Mode	R
-	-	-	Default Mode	L
93	OFC	Orbital Frontal Complex	Frontoparietal	L
97	i6-8	Inferior 6-8 Transitional Area	Frontoparietal	L
103	52	Area 52	Auditory	R
-	-	-	Auditory	L
108	FOP4	Frontal OPercular Area 4	Cingulo-Opercular	L
109	MI	Middle Insular Area	Cingulo-Opercular	L
114	FOP3	Frontal OPercular Area 3	Cingulo-Opercular	L
119	PreS	PreSubiculum	Default Mode	R

120	H	Hippocampus	Default Mode	R
122	PeEc	Perirhinal Ectorhinal Cortex	Ventral-Multimodal	L
126	PHA1	ParaHippocampal Area 1	Default Mode	R
-	-	-	Default Mode	L
127	PHA3	ParaHippocampal Area 3	Dorsal Attention	L
129	STSdp	Area STSd posterior	Language	R
-	-	-	Language	L
130	STSvp	Area STSv posterior	Default Mode	L
131	TGd	Area TG dorsal	Default Mode	L
133	TE1p	Area TE1 posterior	Frontoparietal	L
140	TPOJ2	Area TemporoParietoOccipital Junction 2	Posterior-Multimodal	L
145	IP1	Area IntraParietal 1	Frontoparietal	R
153	VMV1	VentroMedial Visual Area 1	Secondary Visual	L
155	PHA2	ParaHippocampal Area 2	Default Mode	R
-	-	-	Default Mode	L
159	LO3	Area Lateral Occipital 3	Secondary Visual	L
161	31pd	Area 31pd	Default Mode	R
162	31a	Area 31a	Frontoparietal	L
169	FOP5	Area Frontal Opercular 5	Cingulo-Opercular	L
171	p47r	Area posterior 47r	Frontoparietal	L
174	LBelt	Lateral Belt Complex	Auditory	R
179	a32pr	Area anterior 32 prime	Cingulo-Opercular	L

Supplementary Table 6 – Top 60 cortical areas contributing to classification accuracy of DC

Parcel Number	Area Name	Area Description	Resting State Network	Hemisphere
12	55b	Area 55b	Language	R
22	PIT	Posterior InferoTemporal Complex	Secondary Visual	R
-	-	-	Secondary Visual	L
25	PSL	PeriSylvian Language Area	Cingulo-Opercular	L
27	PCV	PreCuneus Visual Area	Posterior-Multimodal	L
28	STV	Superior Temporal Visual Area	Language	R
31	POS1	Parieto-Occipital Sulcus Area 1	Default Mode	R
38	23c	Area 23c	Cingulo-Opercular	R
43	SCEF	Supplementary and Cingulate Eye Field	Cingulo-Opercular	R
50	MIP	Medial IntraParietal Area	Dorsal Attention	L
56	6v	Ventral Area 6	Somatomotor	R
-	-	-	Somatomotor	L
58	33pr	Area 33 prime	Cingulo-Opercular	R
-	-	-	Frontoparietal	L
60	p32pr	Area p32 prime	Cingulo-Opercular	R
-	-	-	Cingulo-Opercular	L
61	a24	Area a24	Default Mode	R
79	IFJa	Area IFJa	Language	R
81	IFSp	Area IFSp	Language	R
83	p9-46v	Area posterior 9-46v	Frontoparietal	L
86	9-46d	Area 9-46d	Cingulo-Opercular	L
92	13l	Area 13l	Frontoparietal	L
93	OFC	Orbital Frontal Complex	Default Mode	R
-	-	-	Frontoparietal	L
99	43	Area 43	Cingulo-Opercular	L
103	52	Area 52	Auditory	R
-	-	-	Auditory	L
105	PFcm	Area PFcm	Cingulo-Opercular	R
106	PoI2	Posterior Insular Area 2	Cingulo-Opercular	L
107	TA2	Area TA2	Auditory	R
110	Pir	Pirform Cortex	Orbito-Affective	R
-	-	-	Orbito-Affective	L
111	AVI	Anterior Ventral Insular Area	Frontoparietal	R
-	-	-	Frontoparietal	L
112	AAIC	Anterior Agranular Insula Complex	Orbito-Affective	R
118	EC	Entorhinal Cortex	Default Mode	R
120	H	Hippocampus	Default Mode	R
-	-	-	Default Mode	L

122	PeEc	Perirhinal Ectorhinal Cortex	Ventral-Multimodal	R
-	-	-	Ventral-Multimodal	L
124	PBelt	ParaBelt Complex	Auditory	R
126	PHA1	ParaHippocampal Area 1	Default Mode	R
127	PHA3	ParaHippocampal Area 3	Dorsal Attention	R
129	STSdp	Area STSd posterior	Language	L
134	TE2a	Area TE2 anterior	Default Mode	L
135	TF	Area TF	Ventral-Multimodal	R
136	TE2p	Area TE2 posterior	Dorsal Attention	R
-	-	-	Dorsal Attention	L
139	TPOJ1	Area TemporoParietoOccipital Junction 1	Language	R
-	-	-	Language	L
140	TPOJ2	Area TemporoParietoOccipital Junction 2	Posterior-Multimodal	R
-	-	-	Posterior-Multimodal	L
147	PFop	Area PF opercular	Cingulo-Opercular	R
166	pOFC	posterior OFC Complex	Orbito-Affective	R
167	PoI1	Area Posterior Insular 1	Cingulo-Opercular	R
169	FOP5	Area Frontal Opercular 5	Cingulo-Opercular	L
170	p10p	Area posterior 10p	Frontoparietal	L
172	TGv	Area TG Ventral	Language	R
173	MBelt	Medial Belt Complex	Auditory	L
178	PI	Para-Insular Area	Cingulo-Opercular	R

Supplementary Figures

Supplementary Figure 1 – Graph measures of LBP and HCs by parcel

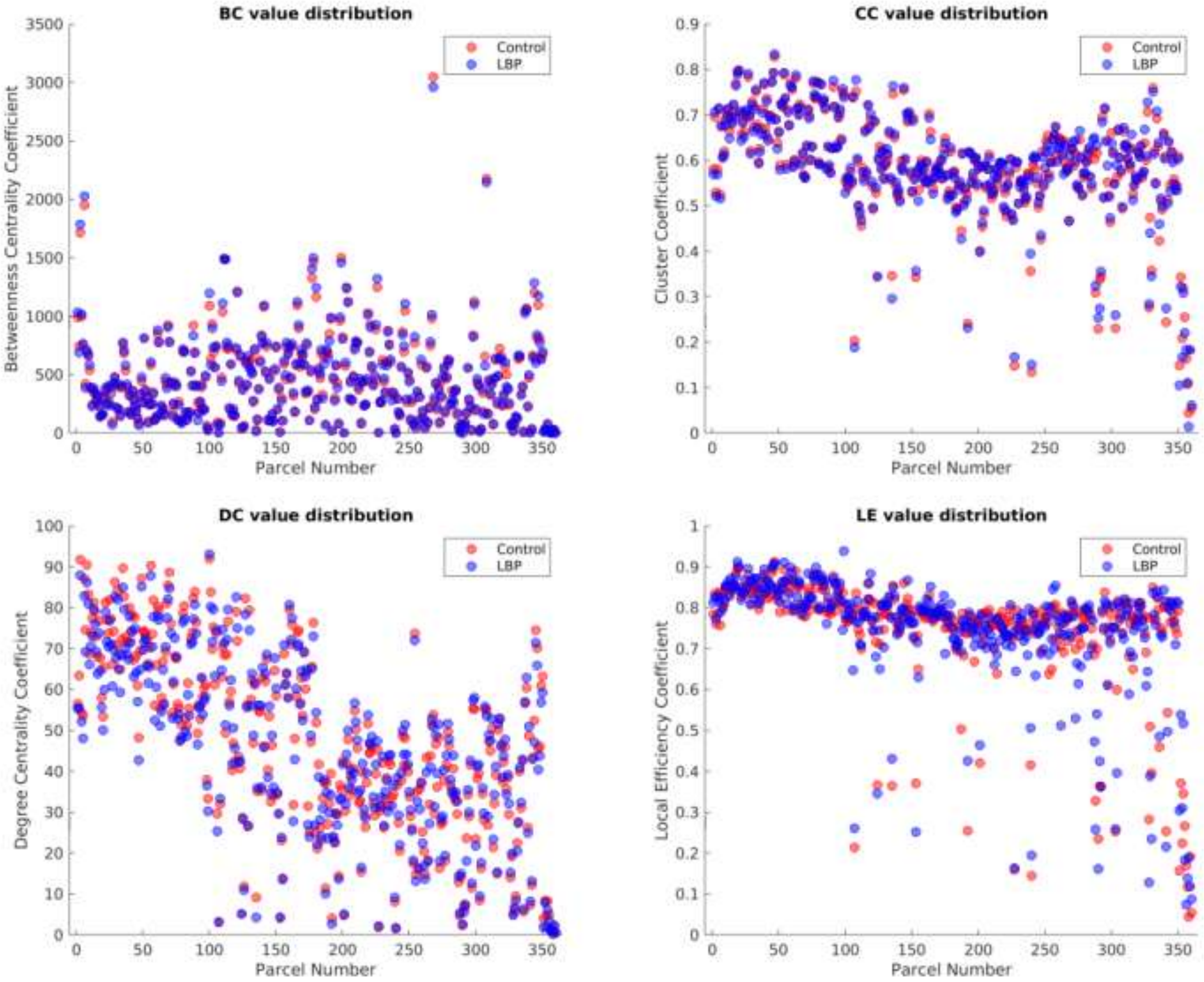

This figure shows the respective graph measures averaged by patient group for low back pain patients and healthy controls by parcel number.

Supplementary Figure 2 – Graph measures of LBP and HCs averaged by network

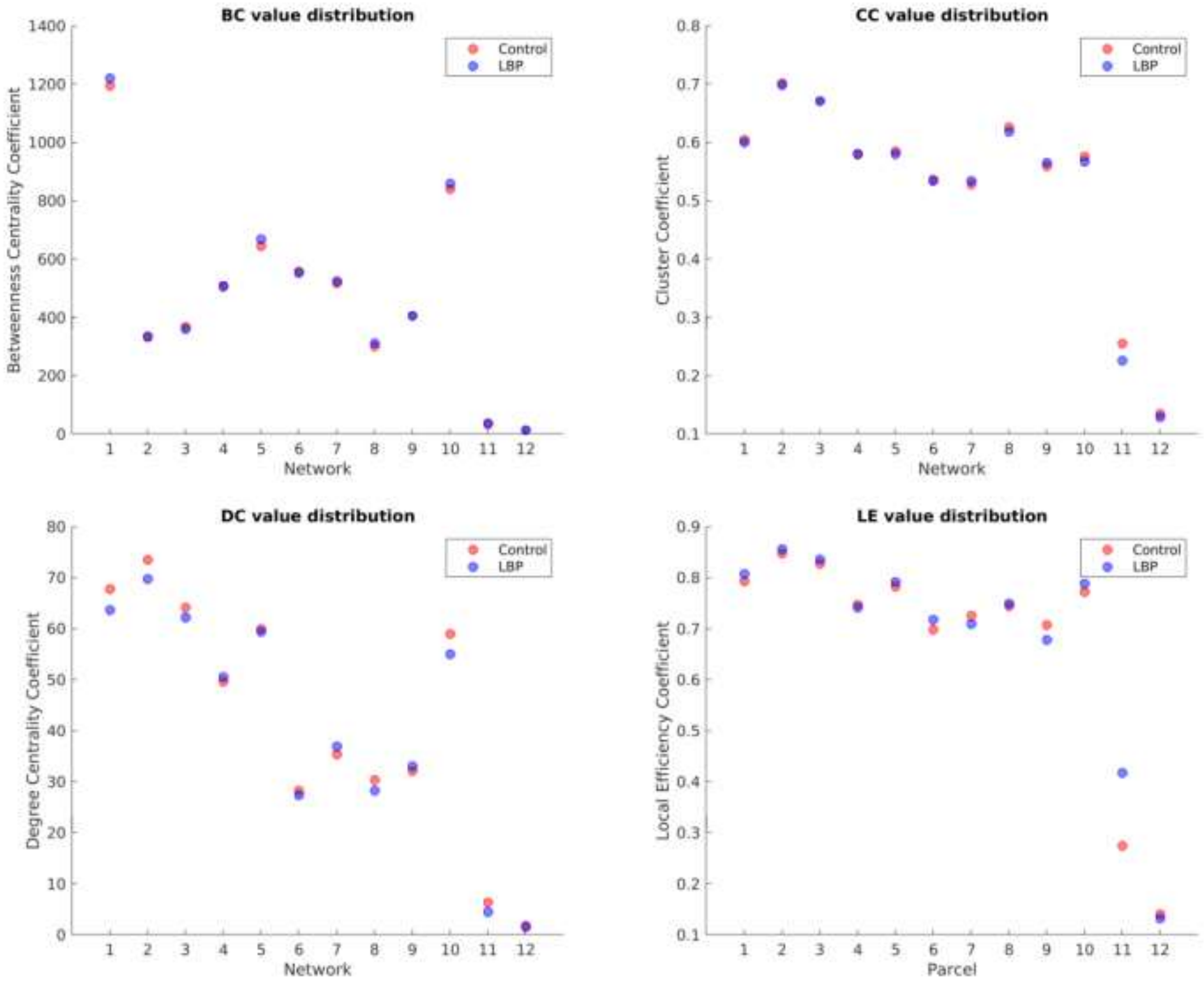

This figure shows the respective graph measures for low back pain patients and healthy controls that have been averaged by patient group and network. Parcels were grouped into one of 12 resting state networks as defined by the Cole-Anticevic network parcellation (Ji et al., 2019). These networks were the primary visual (VIS1), secondary visual (VIS2), auditory (AUD), somatomotor (SOM), cingulo-opercular (CON), default-mode (DMN), dorsal attention (DAN), frontoparietal cognitive control (FPN), posterior multimodal (PML), ventral multimodal (VML), language (LAN), and orbito-affective (OA) networks.

Supplementary Figure 3 – Top 60 cortical areas contributing to classification accuracy of BC

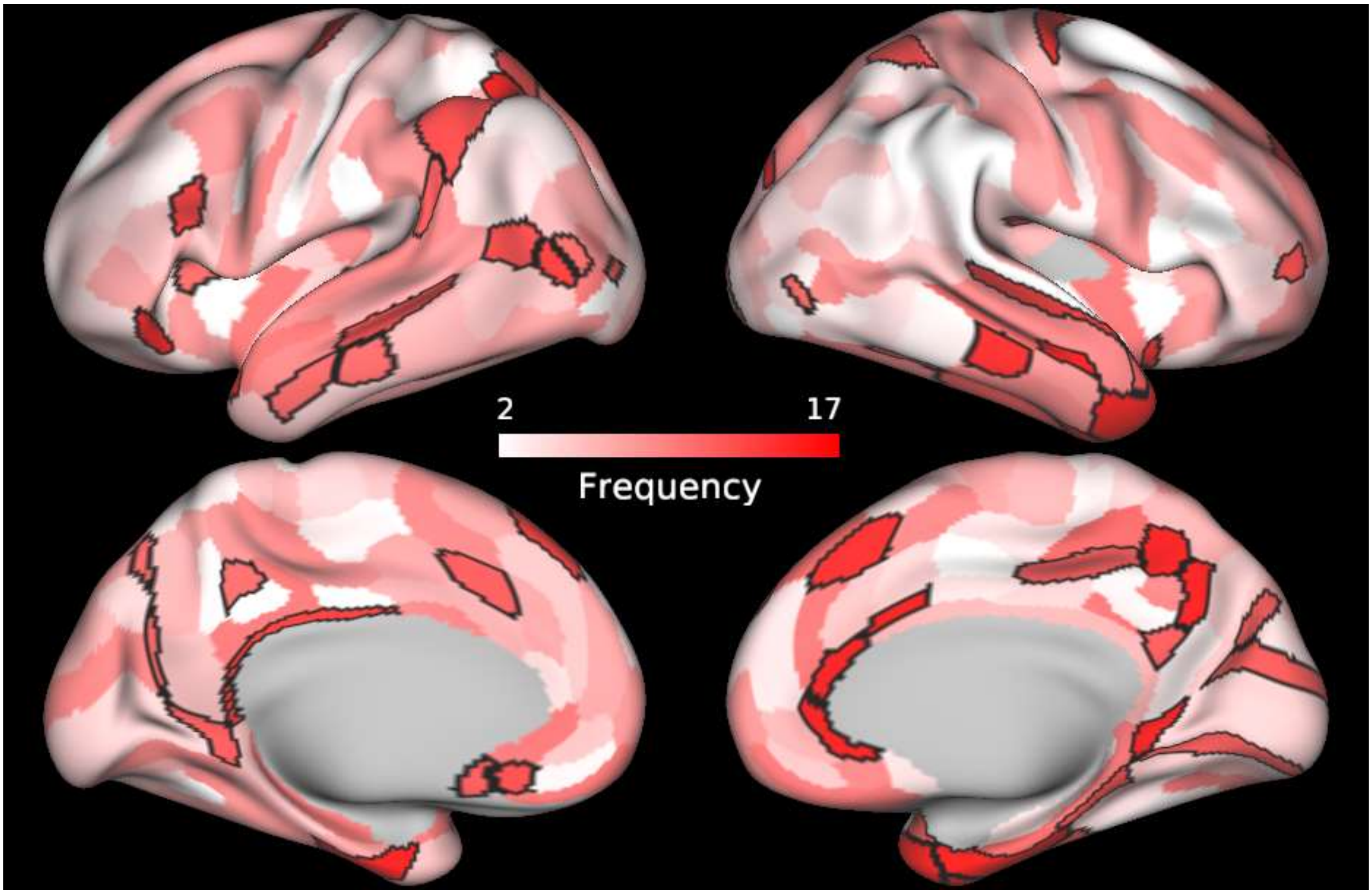

This figure shows the frequency, as a color gradient, of each cortical area that contributed to the classification accuracy of the Enet-subset model when using betweenness centrality data only. The cortical areas outlined in black are the top 60 cortical areas, ranked in descending order by frequency, that contributed to the classification accuracy of this model.

<u>Supplementary Figure 4 – Top 60 cortical areas contributing to classification accuracy of CC</u>

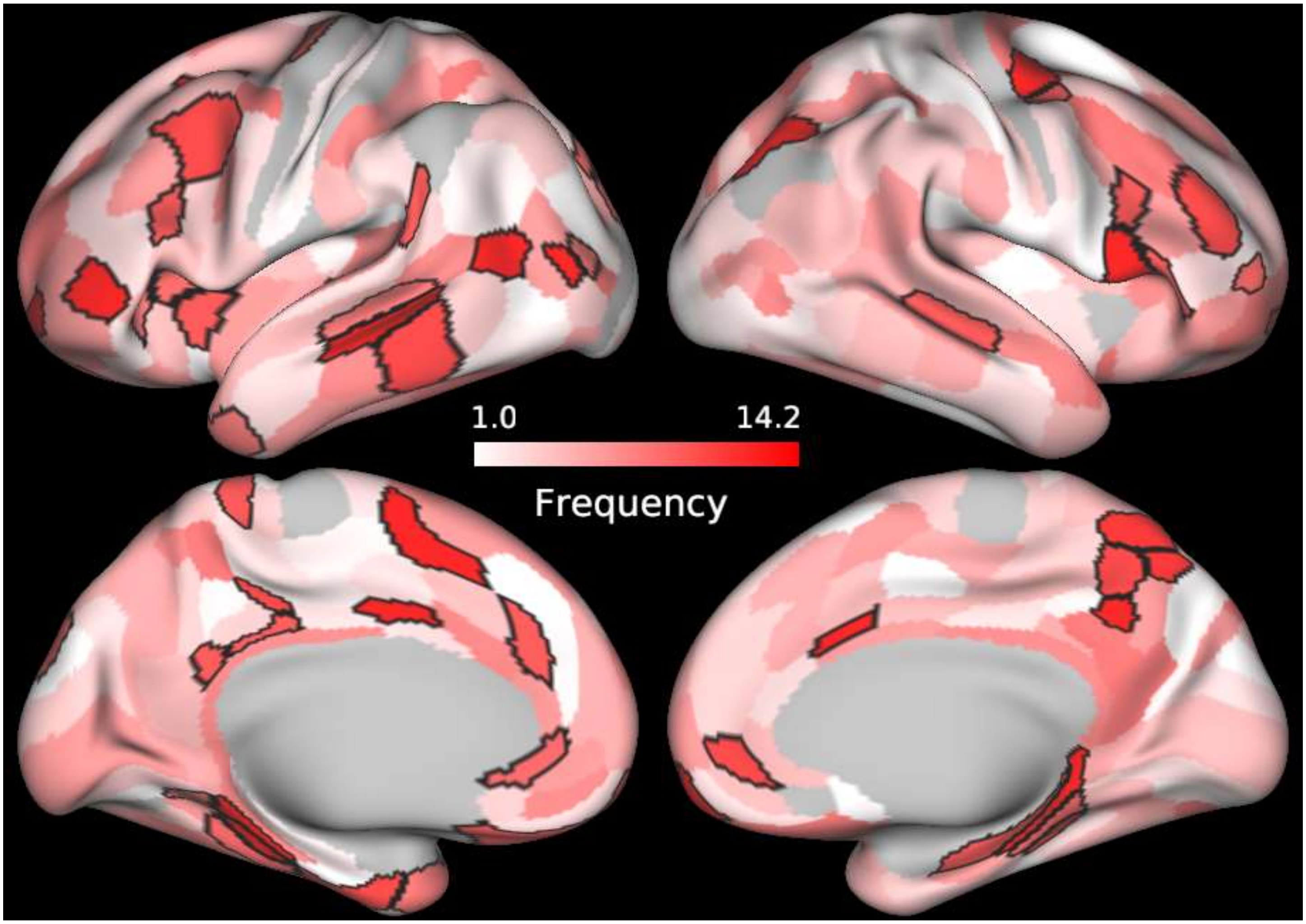

This figure shows the frequency, as a color gradient, of each cortical area that contributed to the classification accuracy of the Enet-subset model when using clustering coefficient data only. The cortical areas outlined in black are the top 60 cortical areas, ranked in descending order by frequency, that contributed to the classification accuracy of this model.

Supplementary Figure 5 – Top 60 cortical areas contributing to classification accuracy of DC

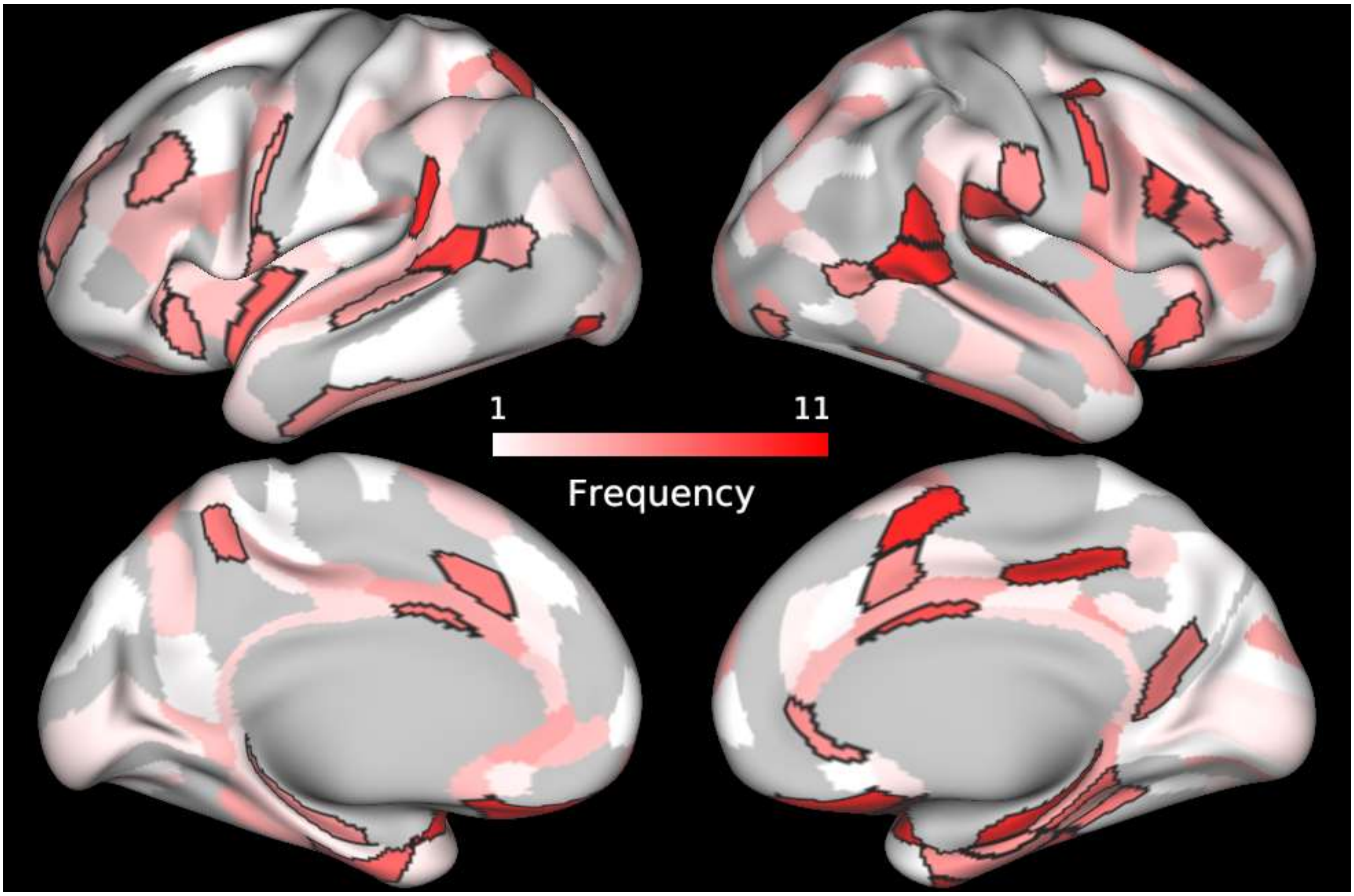

This figure shows the frequency, as a color gradient, of each cortical area that contributed to the classification accuracy of the Enet-subset model when using degree centrality data only. The cortical areas outlined in black are the top 60 cortical areas, ranked in descending order by frequency, that contributed to the classification accuracy of this model.

Supplementary Figure 6 – Bilateral cortical areas from top 60 cortical areas contributing to classification accuracy

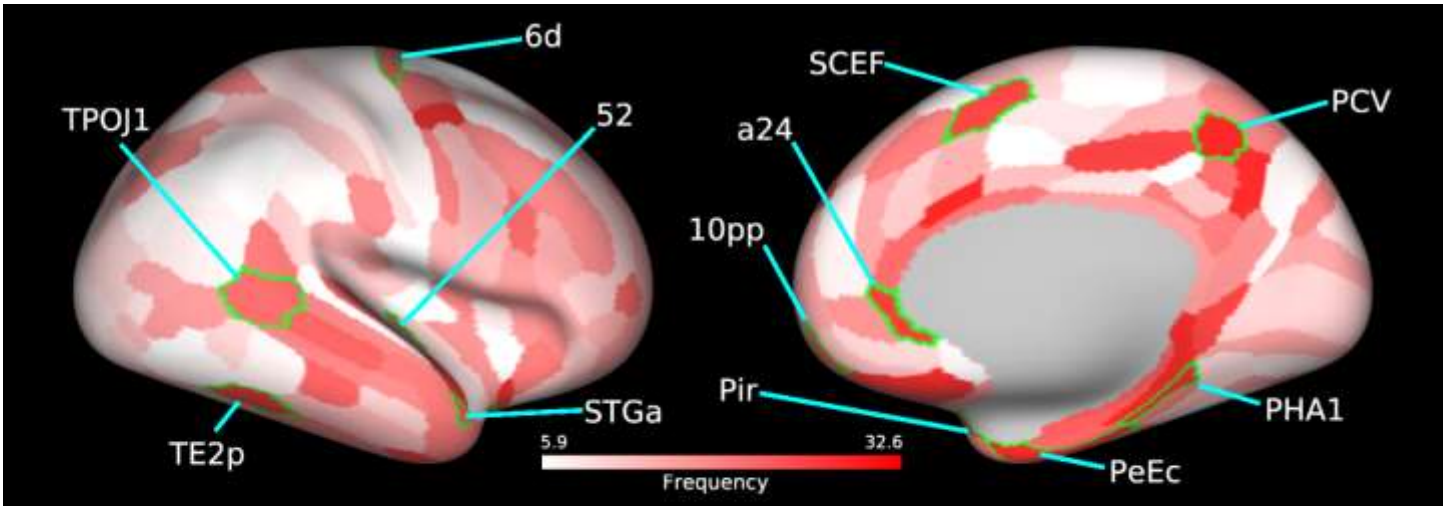

Bilateral cortical regions from the 60 most frequently selected parcels used to train the SVM model using BC+CC+DC and an Enet-subset feature selection method are projected onto a cortical mesh surface of the right hemisphere. Cortical areas are outlined in green and labelled accordingly.
